# Can you BALieve it?

**DOI:** 10.1111/vcp.13204

**Published:** 2022-11-30

**Authors:** Bridget C. Garner

**Affiliations:** ^1^ University of Georgia College of Veterinary Medicine, University of Georgia Athens Georgia USA

**FIGURE 1:**
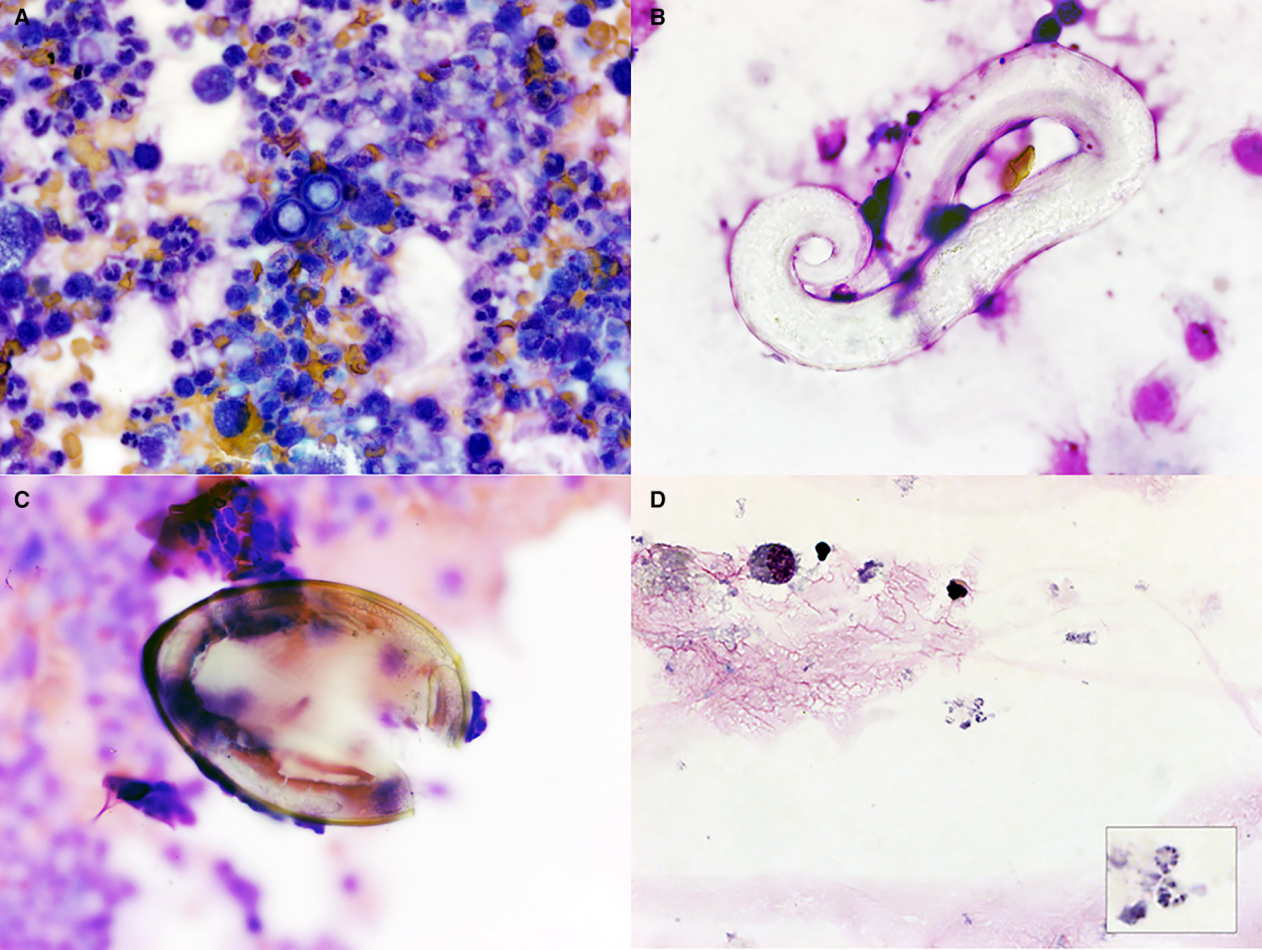
A, BAL from a coughing 12‐year‐old, spayed female, Australian Shepherd dog. Modified Wright's stain, 1000× magnification. Dark blue, thick‐walled yeasts (*Blastomyces dermatitidis*) are surrounded by numerous neutrophils and macrophages. B, BAL from a coughing 1‐year‐old, intact male, Domestic Shorthair cat. Modified Wright's stain, 1000× magnification. A coiled, non‐staining lungworm larva (*Aelurostrongylus abstrusus*) is surrounded by poorly preserved cells. C, BAL from a 2‐year‐old, neutered male, Bassett Hound with a history of hemoptysis. Modified Wright's stain, 1000× magnification. A large, golden ovum with a flat operculum (*Paragonimus kellicott*i) on a background of neutrophils, macrophages, and eosinophils. D, BAL from a 17‐year‐old Thoroughbred gelding with a persistently increased respiratory rate and a history of prolonged steroid administration. Modified Wright's stain, 1000× magnification. There are extracellular cysts (*Pneumocystis* sp.) with approximately eight basophilic bodies arranged in a circle (inset)

Bronchoalveolar lavage (BAL) is routinely used to assess the respiratory tract for abnormalities (Figure 1). While veterinary pathologists are typically focused on the microscopic interpretation of these specimens, let us not ignore the stories beyond clinical pathology.

- Blastomycosis was first described by physician T.C. Gilchrist in 1894. Initially, he believed a protozoan was responsible for his patient's skin lesions, but a tissue biopsy later disproved the initial theory by revealing the budding yeasts.^1^ In the years that followed, blastomycosis became known as “Gilchrist's disease.” Due to the large number of early cases identified in or near Chicago, Illinois, it was also known as “Chicago disease.”^2^
- The Baermann technique is a fecal test based on the active migration of larvae, and it is reported to be the most sensitive test for the detection of *A. abstrusus* infection.^3^ This test was first described in 1917 by the Dutch physician G. K. T. F. Baermann as a simple method of isolating nematodes from soil. The muslin bag initially used to hold the soil allowed pigment and small particles to leak into the water, thereby making it difficult to see any larvae that had also migrated. Since then multiple modifications have been made to this technique, including but not limited to, replacing soil with feces.
- The life cycle for *P. kellicotti* is complicated and includes snail and crustacean intermediate hosts.^4^ The mammalian definitive host usually becomes infected by eating raw or undercooked crustaceans like crabs and crayfish. Crayfish are also known in different regions of the United States as “crawfish,” “crawdads,” and “mudbugs” among other names. They have become a popular festival food and restaurant dish, and in 1983, the crawfish was named the Louisiana state crustacean.^5^
- *Pneumocystis* sp. is a yeast‐like fungus that typically causes respiratory illness in immunocompromised animals and humans. Among dogs, Miniature Dachshunds seem to be predisposed.^6^ A Dachshund named Waldi served as the first official Olympic Summer Games mascot in Munich in 1972, and that year's marathon route corresponded to the mascot's shape.^7^ His cartoon image was multi‐colored to represent the colors of the Olympic rings, rather than one of the many colors and marking combinations actually seen in the breed.
